# Factors Associated with 24-Hour Clinical Outcome of Emergency Patients; a Cohort Study

**DOI:** 10.22037/aaem.v10i1.1590

**Published:** 2022-04-24

**Authors:** Kannika Katsomboon, Siriorn Sindhu, Ketsarin Utriyaprasit, Chukiat Viwatwongkasem

**Affiliations:** 1 DNS candidate, Faculty of Nursing, Mahidol University, Thailand.; 2 Department of Surgical Nursing, Faculty of Nursing, Mahidol University, Thailand; 3 Biostatistics Department, Faculty of Public Health, Mahidol University, Thailand.

**Keywords:** Outcome assessment, health care, clinical decision rules, transportation of patients, patient care management, emergency treatment

## Abstract

**Introduction:**

Pre-hospital and in-hospital emergency management play an important role in quality of care for emergency patients. This prospective cohort study aimed to determine factors associated with the 24-hour clinical outcome of emergency patients.

**Methods:**

The sample included 1,630 patients, randomly selected through multi-stage stratified sampling from 13 hospitals in 13 provinces of Thailand. Data were collected during January-November 2018. Clinical outcome was determined using pre-arrest sign score. Data were analyzed via ordinal multivariate regression analysis.

**Results:**

Factors influencing 24-hour clinical outcome of emergency patients were age (OR: 0.965; 95% CI: 0.96-0.97), having coronary vascular disease (CAD) (OR: 1.41; 95% CI: 1.05-1.88), and severity of illness based on Rapid Emergency Medical Score (REMS) (OR:1.09; 95% CI: 1.05-1.15). Self-transportation and being transported by emergency medical service ambulance with non-advanced life support (EMS-Non-ALS) did not influence clinical outcome when compared to EMS-ALS transport. Being transported from a community hospital increased pre-arrest sign score 1.78 times when compared to EMS-ALS (OR: 1.78; 95% CI: 1.17-2.72). Increased transportation distance increased the risk of poor clinical outcome (OR: 1.01; 95% CI: 1.002-1.011). Length of stay in emergency department (ED-LOS) more than 4 hours (OR: 0.21; 95% CI: 0.15-0.29) and between 2-4 hours (OR: 0.60; 95% CI: 0.47-0.75) decreased the risk of poor clinical outcome when compared to ED-LOS less than 2 hours.

**Conclusion:**

Having CAD, severity of illness, increased transport distance, and ED-LOS less than 2 hours were found to negatively influence 24-hour clinical outcome of emergency patients.

## 1.Introduction:

Pre-hospital emergency medical events are often associated with adverse clinical outcomes such as death or cardiopulmonary arrest. In Bulgaria, overall mortality rate of emergency patients treated in emergency department (ED) was 2.4/ 100000 and 70.9% of deaths happened within 2.3 hour after arrival (1). In Switzerland, the incidence of death in the emergency department (ED) was 2.6/1,000 (2). Patient-related factors, health provider-related factors and health service-related factors have been reported to affect immediate and intermediate outcomes of emergency services. Age is one of the patient-related factors that contributes to mortality of emergency patients (1, 3). Other patient-related factors contributing to mortality among emergency patients include poverty and late arrival to the hospital (1).

Emergency medical service (EMS) systems play a very important role in improving the survival rates. Pre-hospital transport time and distance has been found to influence emergency medical service outcomes (4-6). The roles of mode of transportation to ED on outcomes of emergency patients have been examined in previous studies. In France, no significant association between mode of transportation and all-cause 30-day mortality was noted (4, 7). International literature reported conflicting results on effects of length of ED stay (ED-LOS) on inpatient mortality (8-10). Patient conditions on ED discharge, length of intensive care unit (ICU) stay, and death were used as outcome indicators of emergency management in previous studies (8, 11). These outcomes may not fully reflect the quality of emergency medical service, especially when considering the relationship between pre-hospital emergency management, management in ED, and ongoing management in ICU or inpatient wards following ED discharge. This study aimed to determine factors associated with 24-hour clinical outcome of emergency patients. 

## 2. Methods

***2.1. Study design and settings***

This prospective cohort study was conducted from January to November 2018 in 13 provincial hospitals in Thailand. Data from the National Institute of Emergency Medicine Service (NIEMS, 2017) (12) was used for hospital selection. The provincial hospitals were classified into high volume (i.e., treating > 10,000 critical emergency patients/year), medium volume (i.e., treating between 4,000- 10,000 critical emergency patients/year), and low volume (i.e., treating < 4,000 critical emergency patients/year). There were 13, 33, and 31 high-, medium- and low-volume provincial hospitals, respectively. Subsequent sampling by ratio yielded 3 high-volume hospitals, 5 medium-volume hospitals, and 5 low-volume hospitals. The hospital samples totaled 13. The Institutional Review Board, Faculty of Nursing, Mahidol University (No: IRB -NS2017/23.0409) approved this study for its human research ethics. The patients agreed to participate in this study and provided their consent. To comply with local requirements, additional ethics approvals were also sought from the 13 provincial hospitals before the commencement of the study. 

***2.2 Participants***

The population included emergency patients who were managed in EDs of provincial hospitals across Thailand. The emergency patients in this study referred to those who were triaged as Level-1 and Level-2 based on Emergency Severity Index or ESI (Version 4) (13). Level-1 patients required immediate lifesaving interventions. Level-2 patients were in a high-risk situation, confused or in severe pain or distress. We decided to include patients transported from community hospitals (i.e., inter-hospital transportation) because these patients experienced emergency episodes in their community, sought help in the ED of a nearby community hospital and were transported for definitive emergency care available at provincial hospitals. These patients were, therefore, considered emergency patients using pre-hospital and in-hospital emergency services. 

To be included in the study, patients had to be at least 18 years old and classified as Level- 1 or Level-2 in ED triage based on Emergency Severity Index (Version 4) (13). Patients who were directly admitted to inpatient wards (not through ED), discharged home directly from ED, or transferred to another hospital were excluded. Patients who had been treated as inpatients in community hospitals and referred to EDs at provincial hospitals were also excluded. 

***2.3 Data gathering***

After the patients were stabilized in the ED, research assistants approached them and explained the research objectives and procedures to them. Patients received routine standard care at ED and inpatient wards. Clinical data were documented by nurses or attending physicians in paper and electronic formats. Research assistants then collected these data after 24 hours of hospitalization. The research assistants underwent a 2-hour intensive training on data collection for this project. 

Patients’ sex and age, underlying disease, severity of illness, transportation distance, mode of transportation, and length of stay in ED were evaluated. 

Severity of illness was assessed using Rapid Emergency Medicine Score (REMS) (14). REMS is comprised of six physiological parameters of age, respiratory rate, oxygen saturation, body temperature, systolic blood pressure, pulse rate, and level of consciousness. The REMS score can be used in both trauma and non-trauma patients. Higher REMS is associated with an OR of 1.51 for in-hospital mortality (95% CI 1.45-1.58) (14). 

Mode of transportation referred to how the patient was transported for definitive treatment in the ED of the provincial hospital. In this study, modes of transportation included self-transportation (i.e., a patient was transported by self, family, or bystander), EMS ambulance with advanced life support (EMS-ALS), EMS ambulance with non-advanced life support (EMS-Non-ALS), and inter-hospital transportation. In the former three modes, a patient was directly transported to the ED of a provincial hospital. In the latter mode, a patient first presented to and was treated at a nearby community hospital and thereafter transported to the ED of a provincial hospital. Transportation distance, in kilometer, was determined by the distance between the location where the emergency took place and the provincial hospital. ED-LOS, in minutes, was defined as the interval between the patient’s triage to ED discharge. 

***2.4. Outcome measurement***

The clinical outcome was measured using pre-arrest sign score, which was assessed and documented 24 hours after hospitalization by a nurse in intensive care unit (ICU) or general ward where the patient was treated following ED. The pre-arrest sign, a term widely used in Thailand, is in fact a tool originally known as an Activation Criteria for a Medical Emergency Team (15). The Activation Criteria for a Medical Emergency Team takes into account presenting symptoms, physiological conditions, and laboratory results, which indicate the risk of cardiopulmonary arrest, and hence pre-arrest signs as referred to by Thai clinicians. The score ranges from 0-11. High scores indicate high risk for cardiopulmonary arrest. In this study, the scores were classified into 5 levels:

8-11 points: Severe critical condition/very high risk of cardiac arrest

5-7 points: Critical condition and high risk of cardiac arrest

4 points: Moderate risk of cardiac arrest

2-3 points: Low risk of cardiac arrest

0-1 point: No sign of cardiac arrest

**2.5** ***Statistical analysis***

The formula for survival studies (16) was used for sample size calculation. In the previous study, 17.7% of patients who were treated in ED were placed in intensive care units (17) and were, therefore, considered critically ill. For multi-stratified random sampling, the sample size of 1,630 was needed. To ensure that samples are adequate, 30 more patients were added to the calculated sample size, resulting in the sample size of 1,660. The statistical package for the social sciences for the MS Windows program (SPSS/FW) (Version 21.0) was used for data analysis. Descriptive statistics, univariate regression analysis, and ordinal multivariate logistic regression analysis with a backward technique were performed. A *p* value cut-off of <0.05 and confidence interval of 95% were used.

## 3. Results

***3.1 Baseline characteristics of patients***

**Figure 1:**
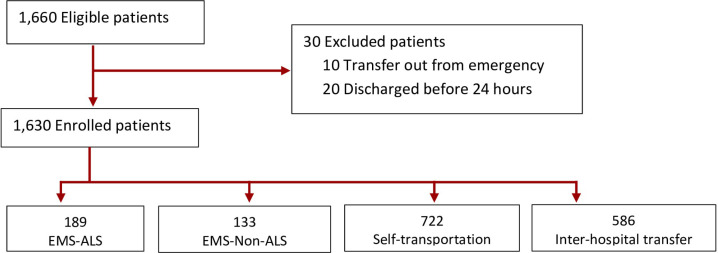
Patient recruitment flowchart. EMS-ALS: Emergency medical service ambulance with advanced life support

A total of 1,630 patients were finally enrolled in the study (Figure 1). The majority were male (57.2%). The mean age was 59.95 + 17.3 (18-98) years. The majority suffered from critical illnesses involving respiratory system (31.2%) and were triaged as ESI Level 2 (66.5%). The most common modes of transportation were self-transportation (44.3%) and inter-hospital transportation (35.9%). Only 11.6% and 8.2% used EMS-ALS and EMS-Non-ALS, respectively. Mean REMS at triage was 6.9 ±3.5 (range 0 -24) points, while mean REMS at discharge from ED was 5.7 ± 3.24 (range 0 -24) points (Table 1).

**3.2 Clinical outcome at 24 hours based on pre-arrest sign**

Based on pre-arrest sign scores, almost half (47.2 %) of the patients had a low level of critical condition after 24 hours of hospitalization. 21.0% and 19.8% had a high and moderate level of critical conditions, respectively. 3.1% were classified as severe (Table1).

**3.3 Associated factors of 24-hour clinical outcome**

Univariate analysis revealed that age, having underlying disease, severity of illness, mode of transportation, transportation distance, and length of stay in ED were associated with pre-arrest sign 24 hours after hospitalization (Table 2). Subsequent ordinal logistic regression revealed that increasing age decreased a risk of developing pre-arrest sign or clinical deterioration 24 hours after hospitalization (OR: 0.965; 95% CI: 0.958-0.973). Patients who had coronary artery disease (CAD) were 1.41 times more likely to experience poor outcome (OR: 1.408; 95% CI: 1.054- 1.882). Every 1-point increase in REMS score at triage increased the risk of clinical deterioration by 1.09 times (OR: 1.097; 95% CI: 1.047-1.150). A 1-point increase in REMS scores at ED discharge, increased the risk of clinical deterioration by 1.28 times (OR: 1.281; 95% CI: 1.212 – 1.354). An increase of 1 kilometer of transportation distance increased the risk of poor outcome by 1.01 times (OR: 1.007; 95% CI: 1.002-1.011). Self-transportation and EMS-Non-ALS transportation produced similar clinical outcomes when compared to EMS-ALS transportation. Patients transported by inter-hospital transfer were 1.78 times more likely to experience deteriorations compared to EMS-ALS (OR: 1.781; 95% CI: 1.167- 2.718). Patients who stayed in ED between 2-4 hours or over 4 hours were less likely to experience clinical deteriorations compared to those who stayed less than 2 hours [(OR: 0.594; 95% CI: 0.468 - 0.753) and (OR: 0.207; 95% CI: 0.146-0.296), respectively].

## 4. Discussion:

Having CAD, severity of illness, increased transport distance, and ED-LOS of less than 2 hours were found to negatively influence 24-hour clinical outcome of emergency patients. Increasing age was found to positively influence the outcome. Modes of transportation (i.e., self-transportation, EMS-ALS, and EMS-Non-ALS) did not influence the outcome. 

We had hypothesized that increasing age of the patient was a risk factor for poor outcome. To our surprise, our result was the opposite. In this study, the older the patients were, the less likely they were at risk of developing pre-arrest sign. The only possible explanation concerns past experience of older persons in relation to how they recognize and respond to warning signs of their underlying illnesses. Emergency medical events experienced by older persons in the past may help them and their family to recognize early changes in their signs and symptoms. 

In the present study, having CAD was a risk factor for poor outcome. CAD has been well-documented as a risk factor of cardiac arrest (18, 19). Patients with existing CAD are more likely to develop cardiopulmonary arrest than those with other pre-existing conditions (19). This finding has several implications for pre-hospital and in-hospital service management. Pre-hospital assessment of emergency patient should include a question about pre-existing CAD. Optimal pre-hospital service arrangements for CAD patients, including equipment and staff with competencies in CAD management, should be considered. 

Severity of illness on ED triage and ED discharge, as determined using REMS, was another predictor of poor clinical outcome. A recent study by Ala et al. (2020) (20) suggested that REMS correlated with mortality at 48 hours and 30 days. In line with emergency service, our finding suggested that REMS may have potential use in pre-hospital, ED, and handover of clinical information for continuous care following ED discharge to ICU or wards. 

Mode of transportation (self-transportation, EMS-Non-ALS, and EMS-ALS) did not make a difference in pre-arrest sign at 24 hours. In Thailand, families and bystanders are familiar with the latter two choices of emergency transportation. When encountering an emergency event, they are required to call the emergency dispatch center or 1669. However, a decision to call or not to call 1669 is influenced by a number of factors such as perceived transport delay and severity of the patients. The patients using self-transportation arrived at the hospital in a shorter time and had a lower level of severity compared to those using EMS transportation and pre-arrest sign was, therefore, not different between self-transportation, EMS-Non-ALS, and EMS-ALS in our study. Our findings are similar to those of a previous study by Seamon, et al. (21). In this study, inter-hospital transportation mode was likely to experience poorer clinical outcome compared to those transported by EMS-ALS. We understand that the increased risk of poor outcome may be related to transportation distance and time. Previous studies reported an increased risk of clinical deteriorations during inter-hospital transfer of critically ill and emergency patients (22).

Transportation distance has shown to increase pre-arrest sign. In long-distance transportation, patients are more likely to experience delay in access to definitive care. These contribute to the increased risk of poor outcome among emergency patients requesting service from a long distance.

The previous national quality standard indicator in Thailand required that ED-LOS is maintained less than 2 hours (23). Previous studies on the effects of ED-LOS on inpatient mortality reported conflicting results. Indian and American studies (9, 24) concluded that ED-LOS had no effect on inpatient mortality rates, whereas an Indian study (9) revealed the effect of longer ED-LOS on higher rate of inpatient death. Our findings, however, suggested that shorter stay (less than 2 hours) was associated with higher risk for developing pre-arrest signs. This can be explained by the fact that the majority (85.5%) of the patients were admitted to general wards compared to only 14.5% admitted to ICU and semi-intensive care unit. Continuous management of emergency and critically ill patients at general wards can be very challenging. These general units are often overworked and understaffed. Our study points out that more time at ED may be beneficial for the patients as it allows clinical conditions to be improved and sustained in the ED and makes it less complex to be managed in general wards. The Ministry of Public Health has very recently increased the ED target length of stay to 2-4 hours (25). 

The results of this study highlight two important processes in emergency medical services in Thailand: safe and early arrival of emergency patients at ED for definitive care and adequate management and stabilization of emergency patients in ED before inpatient admission. 

**Table 1:** Baseline characteristics of study participants

**Variable**	**Value (n=1,630)**
**Age (year) **	
Mean ± SD	59.9 ±17.3
**Sex**	
Male	933 (57.2)
Female	697 (48.2)
**Chief complaint**	
Respiratory	508 (31.2)
Neurological	415 (25.5)
Cardiovascular	310 (19.0)
Cardiopulmonary arrest	142 (8.7)
Trauma	116 (8.5)
Other**	139 (7.1)
**Underlying disease**	
Hypertension	547 (33.6)
Coronary artery disease	309 (19.0)
Diabetes mellitus	148 (9.1)
Chronic obstructive pulmonary disease	121 (7.4)
Epilepsy	28 (1.7)
None	34 (2.1)
**Triage Level**	
ESI level-1	546 (33.5)
ESI level-2	1084 (66.5)
**Transportation distance** **(kilometers)**	
Mean ± SD	28.2 ±31.0 (1-224)
**Mode of transportation**	
Self-transportation	722 (44.3)
Inter-hospital transfer	586 (35.9)
EMS – ALS	189 (11.6)
EMS-Non-ALS	133 (8.2)
**Severity of illness **	
REMS on triage	6.9 ± 3.5 (0-24)
REMS on Discharge	5.7 ± 3.2 (0-24)
**Length of stay in ED (minutes)**	
Mean ± SD	119.36± 131.7 (10-1505)
**Ward Admission **	
General ward	1395(85.5)
Intensive care unit	164 (10.1)
Semi-Intensive care unit	73 (4.4)
**Clinical outcome based on pre-arrest sign**	
No sign of cardiac arrest	146 (9)
Low risk of cardiac arrest	769 (47.2)
Moderate risk of cardiac arrest	322 (19.8)
High risk of cardiac arrest	342 (21.0)
Severe risk of cardiac arrest	51 (3.1)

Data are presented as mean ± standard deviation (SD) or frequency (%). ESI: emergency severity index; REMS: rapid emergency medicine score; EMS-ALS: Emergency medical service ambulance with advanced life support.

**Table 2:** Univariate regression analysis of factors associated with 24-hour clinical outcome of patients admitted to emergency department (n=1,630)

**Variable**	**Level of ** **pre-arrest sign at** ** 24 hours**					**p**
	**No**	**Low**	**Moderate**	**High**	**Severe**	
**Age (year)**						
Mean ± SD	56.88±18.18	59.82±17.43	59.29±17.06	62.11±16.84	60.43±16.38	0.0331
**Sex**						
Male	81 (8.7)	449 (48.1)	194 (20.8)	180 (19.3)	29 (3.1)	0.313
Female	65 (9.3)	320 (45.9)	128 (18.4)	162 (23.2)	22 (3.2)	
**Underlying disease**						
Hypertension	49 (9.0)	250 (45.7)	109 (19.9)	122 (22.3)	17 (3.1)	0.0091
Diabetes mellitus	9 (6.1)	75 (50.7)	32 (21.6)	31 (20.9)	1 (0.7)	
Coronary artery disease	2 (6.5)	17 (54.8)	4 (12.9)	7 (22.6)	1 (3.2)	
Epilepsy	8 (9.5)	44 (52.4)	13 (15.5)	16 (9.0)	3 (3.6)	
COPD	3 (12.5)	11 (45.8)	3 (12.5)	5 (20.8)	2 (8.3)	
None	22 (18.2)	52 (43.0)	21 (17.4)	25 (20.7)	1 (0.8)	
**Severity of illness**						
REMS at triage	5.64 ± 2.73	6.109 ± 3.13	7.37 ± 3.64	8.11 ± 3.55	10.61 ± 4.95	0.0001
REMS at discharge	4.02 ± 2.57	4.94 ± 2.77	6.27 ± 3.17	6.89 ± 3.11	9.96 ± 5.25	0.0001
**Type of transportation**						
Inter-hospital	2 (0.3)	230 (39.2)	150 (25.6)	178 (30.3)	27 (4.6)	0.0001
Self-transportation	109 (15.1)	375 (52.0)	113 (15.7)	116 (16.1)	8 (1.1)	0.0001
EMS-non-ALS	15 (11.0)	68 (50.0)	27 (19.9)	18 (13.2)	8 (5.9)	0.0001
EMS-ALS	20 (10.8)	96 (51.6)	32 (17.2)	30 (16.1)	8 (4.3)	
**Transportation distance (** **Kilometers)**						
Mean ± SD	9.79 ± 7.17	24.75± 28.64	32.39 ± 30.56	38.55 ± 37.07	35.47±4.86	0.0001
**ED-LOS (hours)**						
≤ 2	23 (15.2)	74 (49.0)	15 (9.9)	33 (21.9)	6 (4.0)	0.0001
2-4	45 (12.9)	106 (30.3)	52 (14.9)	119 (34.0)	28 (8.0)	0.001
≥ 4	31 (2.7)	374 (33.1)	197 (17.4)	417 (36.9)	110 (9.7)	

**Table 3:** Ordinal logistic regression analysis of 24-hour clinical outcome-associated factors in patients admitted to emergency department (n=1,630)

**Factor**	**Estimate**	**Wald**	**OR**	**95% CI**		***p***
				**Lower**	**Upper**	
**Age**	-0.035	0.004	0.965	0.958	0.973	<.001*
**Underlying disease**						
Coronary artery disease	0.342	5.347	1.408	1.054	1.882	.021*
None	ref					
**Severity of illness**						
REMS at triage	.093	15.072	1.097	1.047	1.150	<.001*
REMS at Discharge	.248	77.081	1.281	1.212	1.354	<.001*
**Transportation distance**	0.007	8.224	1.007	1.002	1.011	0.004*
**Mode of transportation **						
Inter-hospital transfer	0.577	7.148	1.781	1.167	2.718	0.008*
Self-transportation	-0.251	2.356	0.778	0.565	1.072	0.125
EMS-Non-ALS	0.085	0.147	1.089	0.705	1.681	0.701
EMS-ALS	ref					
**Length of stay in emergency department**						
≥ 4 hours	-1.573	75.669	0.207	0.146	0.296	<.001*
2-4 hours	-.521	18.515	0.594	0.468	0.753	<.001*
≤ 2 hours	ref					

CI: confidence interval; OR: odds ratio; REMS: rapid emergency medicine score; EMS-ALS: Emergency medical service ambulance with advanced life support.

## 5. Conclusions:

Having CAD, severity of illness, increased transport distance, and shorter ED-LOS of less than 2 hours were found to negatively influence 24-hour clinical outcome of emergency patients. Increasing age was found to positively influence the outcome. Modes of transportation (i.e., self-transportation, EMS-ALS, and EMS-Non-ALS) did not influence the outcome. 

## 6. Declarations

### 6.1 Acknowledgements

We thank all the participants in this study as well as staff and health professionals for their facilitation during data collection.

### 6.2 Authors’ contributions

K.K. and S.S.; Contributed to conception, study design. K.K.; Contributed to data gathering and evaluation. K.K., S.S and V.C.; Contributed to statistical analysis, and interpretation of data. K.K.; Drafted the manuscript, which was revised by K.K. and S.S. All authors read and approved the final manuscript.

### 6.3. Authors ORCIDs

Kannika Katsomboon:0000-0003-0597-0092

Siriorn Sindhu: 0000-0001-9326-757X

Chukiat Viwatwongkasem: 0000-0003-4918-2717

### 7.4 Funding/Support

Research Assistantships, Mahidol University

### 6.5. Conflict of interest

The authors declared that they have no potential conflicts of interests with respect to the research, authorship, and/or publication of this article.
